# Aminopeptidase N (CD13) Is Involved in Phagocytic Processes in Human Dendritic Cells and Macrophages

**DOI:** 10.1155/2013/562984

**Published:** 2013-08-26

**Authors:** Mónica I. Villaseñor-Cardoso, Dulce A. Frausto-Del-Río, Enrique Ortega

**Affiliations:** Departamento de Inmunología, Instituto de Investigaciones Biomédicas, Universidad Nacional Autónoma de México, Apartado Postal 70228, Ciudad Universitaria, 04510 México, DF, Mexico

## Abstract

Aminopeptidase N (APN or CD13) is a membrane ectopeptidase expressed by many cell types, including myelomonocytic lineage cells: monocytes, macrophages, and dendritic cells. CD13 is known to regulate the biological activity of various peptides by proteolysis, and it has been proposed that CD13 also participates in several functions such as angiogenesis, cell adhesion, metastasis, and tumor invasion. We had previously reported that, in human monocytes and macrophages, CD13 modulates the phagocytosis mediated by receptors for the Fc portion of IgG antibodies (Fc**γ**Rs). In this work, we analyzed the possible interaction of CD13 with other phagocytic receptors. We found out that the cross-linking of CD13 positively modulates the phagocytosis mediated by receptors of the innate immune system, since a significant increase in the phagocytosis of zymosan particles or heat-killed *E. coli* was observed when CD13 was cross-linked using anti-CD13 antibodies, in both macrophages and dendritic cells. Also, we observed that, during the phagocytosis of zymosan, CD13 redistributes and is internalized into the phagosome. These findings suggest that, besides its known functions, CD13 participates in phagocytic processes in dendritic cells and macrophages.

## 1. Introduction

Aminopeptidase N/CD13 (EC 3.4.11.2) is a transmembrane ectoenzyme with a short (8–10 amino acids) N-terminal cytoplasmic portion and a large extracellular domain containing the active site. CD13 is expressed on several tissues and cell types. Within the hematopoietic system, it is expressed on stem cells and during most developmental stages of myeloid cells: monocytes, macrophages, basophils, neutrophils, eosinophils [1], and dendritic cells [2, 3]. Although its peptidase activity has been known for many years, recent evidence has shown that CD13 is a multifunctional protein with at least three types of activities: (a) peptide cleavage, (b) endocytosis, and (c) signaling. Due to these activities, CD13 has been suggested to participate in several functions, such as angiogenesis, tumor cell invasion, and metastasis [4–6], as well as functions related to defense against pathogens, such as antigen presentation and phagocytosis, among others (reviewed in [7]).

Phagocytosis involves the engulfment of large particles, typically 1 *μ*m or larger, including particles as diverse as inert beads, apoptotic cells, and microbes [8]. Phagocytosis is a multistep process initiated by the binding of the particle to the cell, mediated by cell surface receptors recognizing the particle, either directly or indirectly—through opsonins. Engagement of the phagocytic receptors triggers signaling cascades leading to cytoskeleton rearrangement, membrane remodeling, and, ultimately, particle engulfment [9]. Phagocytosis is an important function of monocytes, macrophages, and dendritic cells (DCs), not only for clearance and destruction of pathogenic microorganisms, but also for the generation of peptides to be presented to T cells and for triggering the release of cytokines that orchestrate inflammatory reactions and modulate adaptive immune responses.

In mammals, professional phagocytes such as macrophages, DCs, and granulocytes continually sense and sample the extracellular environment. This constant surveillance requires a set of different cell surface receptors in order to recognize and respond to infectious and noninfectious injury [10]. Many of these receptors are able to mediate phagocytosis. Previous reports have suggested that CD13 might participate in phagocytosis. The first study suggesting a correlation between CD13 expression and phagocytic capacity was reported by Tokuda and Levy [11]. They found that cells with high phagocytic capacity express more than twice the level of CD13 than cells with lower phagocytic capacity. Later, we documented the participation of CD13 in IgG-mediated phagocytosis in human peripheral blood monocytes and in U-937 promonocytic cells [12]. We showed that during Fc*γ*RI-mediated phagocytosis, CD13 redistributes to the phagocytic cup and is internalized into the phagosomes. Interestingly, particles were ingested more efficiently when they bound to the phagocyte through Fc*γ*RI and CD13 simultaneously than when they interacted through the Fc*γ*RI only. Additionally, Fc*γ*RI and CD13 colocalized in zones of cellular polarization and coredistributed after aggregation of one or the other [12]. These results established that CD13 modulates phagocytosis by Fc*γ*Rs and participates in the phagocytosis of IgG-opsonized particles, but whether it can also cooperate with nonopsonic receptors is still unknown. A possible role of CD13 in phagocytosis mediated by the mannose receptor has been suggested [13]. On the other hand, Winnicka et al. [14] reported that lack of CD13 in mouse macrophages did not affect Fc*γ*R-mediated phagocytosis or scavenger receptor-mediated endocytosis.

In this study, we investigated the possible cooperation of CD13 with innate immunity receptors in mediating phagocytosis and cytokine production in human monocyte-derived dendritic cells (MD-DCs) and macrophages (MD-Ms). Our results showed that the cross-linking of CD13 positively modulates the phagocytosis of zymosan and bacteria and that CD13 is internalized to the phagosome during the phagocytosis of zymosan, suggesting that CD13 plays a role in phagocytic processes in dendritic cells and macrophages.

## 2. Materials and Methods

### 2.1. Reagents and Antibodies

Fetal bovine serum (FBS), RPMI 1640 medium, MEM sodium pyruvate solution, and MEM nonessential amino acids solution were purchased from GIBCO Laboratories (Grand Island, NY). Bovine serum albumin (BSA) was from Sigma (St. Louis, MO). Ficoll-Paque PLUS was purchased from GE Healthcare Bio-Science (Uppsala, Sweden). Murine monoclonal anti-human CD13 (clone 452, IgG1) was purified in our laboratory from supernatant of the corresponding hybridoma obtained from Dr. Meenhard Herlyn (Wistar Institute of Anatomy and Biology, Philadelphia, PA). F(ab′)_2_ fragments of mAb 452 were prepared with immobilized Ficin (Pierce, Rockford, IL) following the manufacturer's instructions. Murine monoclonal anti-human Fc*γ*RI (mAb32.2) was purified from supernatants of the corresponding hybridoma obtained from the ATCC. Fab fragments were prepared from the purified antibody with Immobilized Pepsin (Pierce). F(ab′)_2_ fragments of goat anti-mouse IgG were bought from Jackson Immunoresearch (West Grove, PA). Antibodies specific for human CD1a and CD14 were purchased from BD Pharmingen (San Jose, CA). Recombinant human IL-4 and GM-CSF, as well as ELISA kits for IL-6, IL-10, IL-12, and TNF-*α*, were purchased from Peprotech Inc. (Rocky Hill, NJ). Peptidoglycan from *Staphylococcus aureus*, as well as carboxyfluorescein-succinimidyl ester (CFSE), was bought from Sigma-Aldrich (Switzerland). Sulfo-NHS-Biotin was purchased from Thermo Scientific Pierce Inc. (Rockford, IL). FITC was from Sigma-Aldrich (Switzerland). Streptavidin was from Calbiochem (San Diego, CA). Streptavidin-Texas Red was obtained from Zymed (San Francisco, CA). Perm/Wash solution for intracellular staining was purchased from BD Biosciences (San Diego, CA). Goat anti-rabbit IgG F(ab′)_2_ and rabbit anti-goat IgG (H+L)-FITC were bought from Jackson Immunoresearch (West Grove, PA). Zymosan was purchased from Sigma-Aldrich (Switzerland). Antifade solution with DAPI was from Dako (Denmark).

### 2.2. Human Monocyte-Derived Macrophages (MD-Ms) and Monocyte-Derived Dendritic Cells (MD-DCs)

Buffy coats from healthy male donors were provided by the Central Blood bank of the Centro Médico Nacional Siglo XXI, IMSS, which also approved their use for these experiments. All experiments were performed following the Ethical Guidelines of the Instituto de Investigaciones Biomédicas, UNAM. All data was analyzed anonymously. 

Human peripheral blood mononuclear cells (PBMCs) were isolated by density gradient centrifugation from buffy coats using Ficoll-Paque PLUS (GE Healthcare, Sweden). PBMCs were washed four times with PBS pH 7.4, and cultured in RMPI-1640 medium supplemented with 10% (v/v) heat-inactivated autologous plasma-derived serum, 1 mM MEM sodium pyruvate solution, 2 mM MEM nonessential amino acids solution, 0.1 mM L-glutamine, 100 U/mL penicillin, and 100 *μ*g/mL streptomycin, for 30 minutes at 37°C in order to allow monocytes to adhere to the plastic plate. Nonadherent cells were eliminated by washes, and adherent cells—enriched for monocytes—were cultured for 7 days in RPMI-1640 medium supplemented with 10% (v/v) heat-inactivated FBS in humidified atmosphere at 37°C with 5% CO_2_. The resulting MD-Ms were harvested by mechanical scrapping and used for experiments.

*Monocyte Derived-Dendritic Cells*. MD-DCs were differentiated from monocyte-enriched fractions. After the adherence step (see above) monocytes were cultured in 6-well plates (Nunclon, Roskilde, Denmark) with 4 mL of RPMI 1640 medium supplemented with 10% FBS, 1 mM sodium pyruvate, 0.1 mM MEM nonessential amino acid solution, 0.1 mM L-glutamine, 100 U/mL penicillin, 100 *μ*g/mL streptomycin, 1000 U/mL IL-4, and 800 U/mL GM-CSF (Peprotech Inc., Rocky Hill, NJ) for seven days. On day 2, 2 mL of medium were removed and replaced with fresh medium. On day 7, nonadherent cells were collected and used for the experiments. Membrane expression of CD1a and CD14 by MD-Ms and MD-DCs was evaluated by direct immunofluorescence staining and cytofluorometry in a FACscan cytometer (Becton Dickinson). 

### 2.3. Effect of CD13 Cross-Linking on Phagocytosis of Zymosan Particles or *E. coli*

FITC zymosan and FITC conjugated heat-killed *E. coli* were prepared as described by Bassoe [15]. Briefly, zymosan (500 *μ*g) (Sigma-Aldrich, Switzerland) was incubated with 0.05 mg/mL of fluorescein isothiocyanate (FITC) (Sigma-Aldrich, Switzerland) in Dulbecco's phosphate-buffered saline solution (DPBS) for 30 min at 37°C and washed thrice with DPBS. For FITC labeling of *E. coli*, 10 mL of a suspension of heat-killed *E. coli* (OD at 620 nm = 1.0) was pelleted and resuspended in 1 mL of 0.9% NaCl, mixing until no clumps were visible. Bacteria were diluted in 9 mL ice-cold 70% ethanol and incubated 30 min at 0°C. Bacteria were washed thrice with 0.9% NaCl and resuspended in 10 mL. Three mL of a FITC solution (0.1 mg/mL in BHI) were added, and the mixture was incubated 30 min at 37°C. After three washes, bacteria were resuspended in Sorensen's buffer and adjusted to OD_620 nm_ = 1.0.

Cells (MD-Ms or MD-DCs) were incubated with 10 *μ*g F(ab′)_2_ fragments of anti-CD13 mAb, for 30 min at 4°C. After washing, CD13 cross-linking was induced by addition of F(ab′)_2_ fragments of goat anti-mouse IgG antibodies (0.75 *μ*g) and mixed with 20 *μ*g FITC-zymosan or 12 *μ*L FITC-*E. coli* suspension. Phagocytosis was allowed to proceed for 30 minutes (MD-Ms) or 1 h (MD-DCs) at 37°C. Negative controls were prepared in identical conditions but incubated at 4°C. Cell suspensions were washed with ice-cold PBS so as to stop phagocytosis and eliminate free zymosan and bacteria. Samples were fixed with PFA 1% and kept at 4°C until analysis in a FACscan cytometer with CellQuest software (Becton Dickinson). Each sample was analyzed both before and immediately after addition of Trypan Blue (1 mg/mL, pH 4.5), in order to quench the fluorescence of nonengulfed particles. Results are indicated as fluorescence-positive cells percentage and/or phagocytic index. The latter was calculated as the product of the percentage of positive cells multiplied by the geometric mean of the fluorescence intensity (GMFI) of the positive population. Negative controls (cells incubated at 4°C with labeled particles) were used to set the negative population.

### 2.4. CD13-Mediated Phagocytosis of SRBCs

Sheep red blood cells (SRBC) were maintained in Alsever's solution for up to 14 days. Prior to the assay, SRBCs (1.2 × 10^9^) were stained with 10 mM carboxyfluorescein-succinimidyl ester (CFSE) (Sigma-Aldrich, Switzerland) for 30 minutes at 37°C in the dark. After been washed, the erythrocyte membrane was derivatized with biotin by incubation with 25 *μ*g sulfo-NHS-Biotin (Thermo Scientific, Rockford, IL) for 20 minutes on ice. Erythrocytes were washed with PBS, incubated with 62.5 *μ*g/500 *μ*L streptavidin (Calbiochem, San Diego, CA) for 20 min at 4°C, washed, and incubated with biotinylated F(ab′)_2_ fragments of goat anti-mouse IgG antibodies (Jackson Immunoresearch, West Grove, PA) for 30 minutes on ice. For the phagocytosis assay, MD-DCs (2.5 × 10^5^ cells in 250 *μ*L) were incubated with 2.5 *μ*g F(ab′)_2_ fragments of either anti-CD13 mAb or anti-Fc*γ*RI mAb, for 30 min at 4°C. After been washed, cells were mixed with 5 × 10^6^ SRBCs covered with F(ab′)_2_ fragments of goat anti-mouse antibodies (as previously mentioned) and incubated for 1 h at 37°C. Identical samples were incubated at 4°C in order to be used as controls. Noninternalized erythrocytes were lysed by incubation with ice-cold distilled water for 1 min, after which isotonicity was restored, and the cells were washed and fixed with 120 *μ*L of 1% PFA. Phagocytosis was analyzed using flow cytometry. Results are indicated as percentage of fluorescence-positive cells and/or phagocytic index. The latter was calculated as the product of the percentage of positive cells multiplied by the geometric mean of the fluorescence intensity of the positive population. Negative controls (cells incubated with erythrocytes at 4°C) were used to set the negative population. 

### 2.5. Confocal Microscopy

Zymosan particles were labeled with biotin by incubation with biotin-hydroxysuccinimide ester for 30 min at 4°C. After washing, they were labeled with Streptavidin-Texas Red (Zymed, San Francisco, CA) for 30 min at 4°C. Labeled particles were washed six times with PBS and were kept in aliquots at −70°C until used. MD-Ms (1 × 10^6^) were incubated with 20 *μ*g zymosan particles for 20 minutes at either 37 or 4°C. After this incubation, free particles were eliminated by washing with ice-cold PBS. Cells were fixed with 4% PFA for 20 min, centrifuged, incubated in Permwash solution (BD Bioscience, San Diego, CA) for 15 min, and washed. Nonspecific binding was blocked through incubation for 30 min with 3% BSA. After washing, 30 *μ*g anti-CD13 mAb 452 was added for 30 min at 4°C, followed by 30 min incubation with FITC-labeled goat anti-mouse IgG. After washing, cells were mounted in glass slides using antifade solution with DAPI (DAKO, Denmark) and analyzed in a Zeiss LSM5 confocal microscope.

### 2.6. Cytokine Secretion

In order to evaluate the ability of CD13 cross-linking to induce cytokine secretion and the effect of CD13 cross-linking on the production of cytokines induced by TLR2 activation, MD-DCs or MD-Ms (1 × 10^6^) were stimulated with different antibodies (10 *μ*g of complete anti-CD13 mAb 452, 10 *μ*g F(ab′)_2_ fragments of anti-CD13 mAb followed by cross-linking with F(ab′)_2_ fragments of goat anti-mouse IgG, or 10 *μ*g nonspecific IgG antibodies) in 1 mL RPMI 1640 medium supplemented with 10% FBS. TLR 2-mediated cytokine secretion was induced with 10 *μ*g Peptidoglycan (PGN) from *Staphylococcus aureus* (Sigma-Aldrich, Switzerland). After incubation for 18 h with the stimuli, cell-free supernatants were harvested, and IL-6, IL-10, IL-12, and TNF-*α* concentrations were determined by standard sandwich enzyme-linked immunosorbent assay following the manufacturer's instructions (Peprotech Inc., Rocky Hill, NJ).

### 2.7. Statistical Analysis

Data were analyzed using paired, two-tailed Student's *t*-test. Differences were considered significant at *P* < 0.05. Data are expressed as mean ± SD.

## 3. Results

### 3.1. CD13 Cross-Linking Increases the Phagocytosis of Zymosan Particles by Human Monocyte-Derived Dendritic Cells and Macrophages

**Figure 1:**
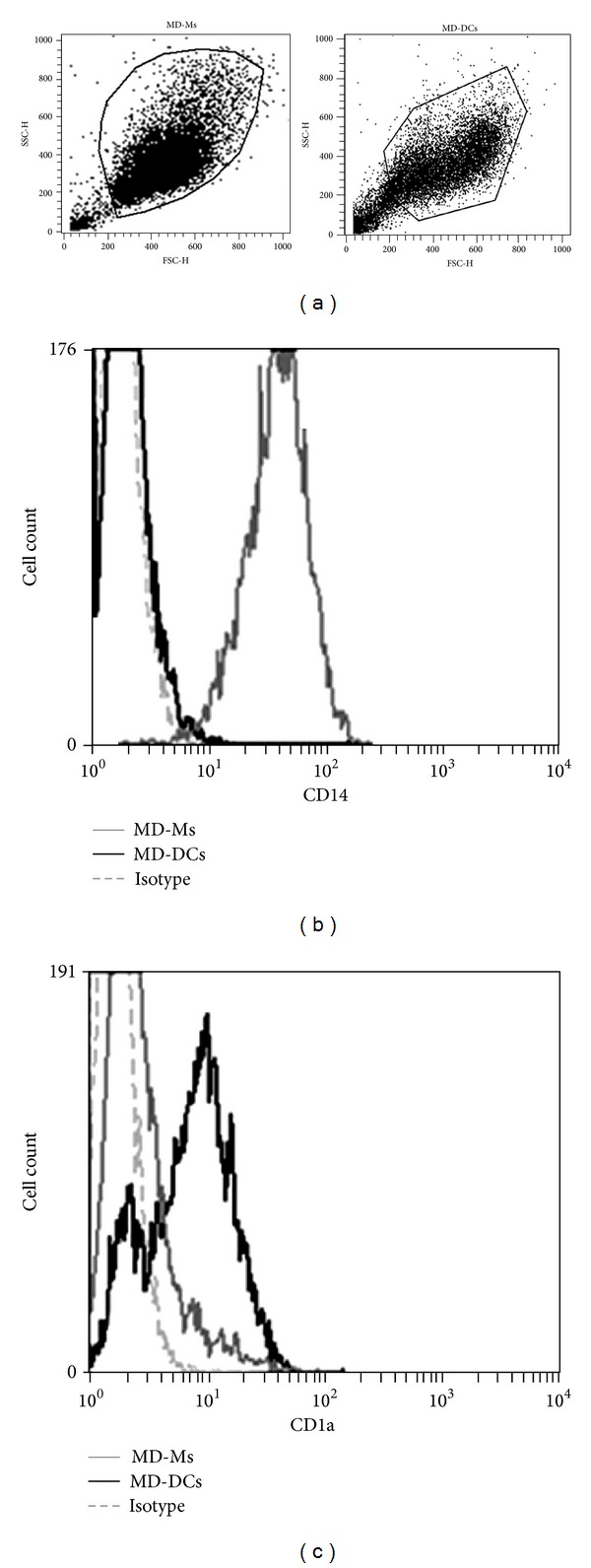
Phenotypic differences in human monocyte-derived macrophages (MD-Ms) and monocyte-derived DCs (MD-DCs). Freshly isolated human monocytes were incubated in RPMI complete medium containing 1000 U/mL IL-4 and 800 U/mL GM-CSF (for MD-DCs) or complete RPMI medium without cytokines (for MD-Ms) for 7 days. The dot plots (a) show the populations of MD-Ms and MD-DCs obtained after 7 days in culture. Expression of CD14 (b) and CD1a (c) was evaluated using flow cytometry. Histograms correspond to MD-Ms (gray line), MD-DCs (black line), and isotype control (dashed line). MD-Ms were 95% CD14+ and 6% CD1a+. MD-DCs were 7% CD14+ and 84% CD1a+.

Human monocytes obtained from PBMCs of healthy donors were differentiated to MD-DCs by their culture* in vitro* with IL-4 plus GM-CSF for seven days. The acquisition of a dendritic cell phenotype was corroborated by a marked decrease in expression of CD14 and the expression of CD1a by more than 90% of the cells [16]. In contrast, MD-Ms retained CD14 expression and did not express CD1a (Figure 1).

We have previously reported that, in human monocytes, the phagocytosis of particles (SRBCs) interacting with the cell through both CD13 and Fc*γ*RI was higher than the phagocytosis of particles interacting through Fc*γ*RI only [12]. In order to evaluate whether intracellular signals induced through CD13 could affect phagocytosis mediated through other phagocytic receptors, we evaluated the effect of CD13 cross-linking with specific antibodies on the phagocytosis of FITC-labeled nonopsonized zymosan particles. Thus, MD-DCs or MD-Ms were incubated at 4°C with or without F(ab′)_2_ fragments of anti-CD13 mAb 452, and, after washing, cross-linking was induced with F(ab′)_2_ fragments of goat anti-mouse IgG, added simultaneously with zymosan-FITC particles. After incubation for 60 min (for MD-DCs) or 30 min (for MD-Ms) at 37°C, phagocytosis was evaluated using flow cytometry. Both MD-DCs and MD-Ms efficiently internalized zymosan particles. The percentage of cells with internalized zymosan was 54.4 ± 15% (*n* = 8) for MD-DCs and 57.1 ± 15% (*n* = 6) for MD-Ms. Simultaneous CD13 cross-linking resulted in a statistically significant increase in the percentage of cells that internalized zymosan, to 64.7 ± 11% for MD-DCs and to 75.1 ± 12% for MD-Ms. Incubation of MD-DCs or MD-Ms with F(ab′)_2_ fragments of anti-CD13 mAb 452, with no cross-linking or with F(ab′)_2_ fragments of goat anti-mouse antibodies only, had no effect on the phagocytosis of zymosan (not shown).

**Figure 2:**
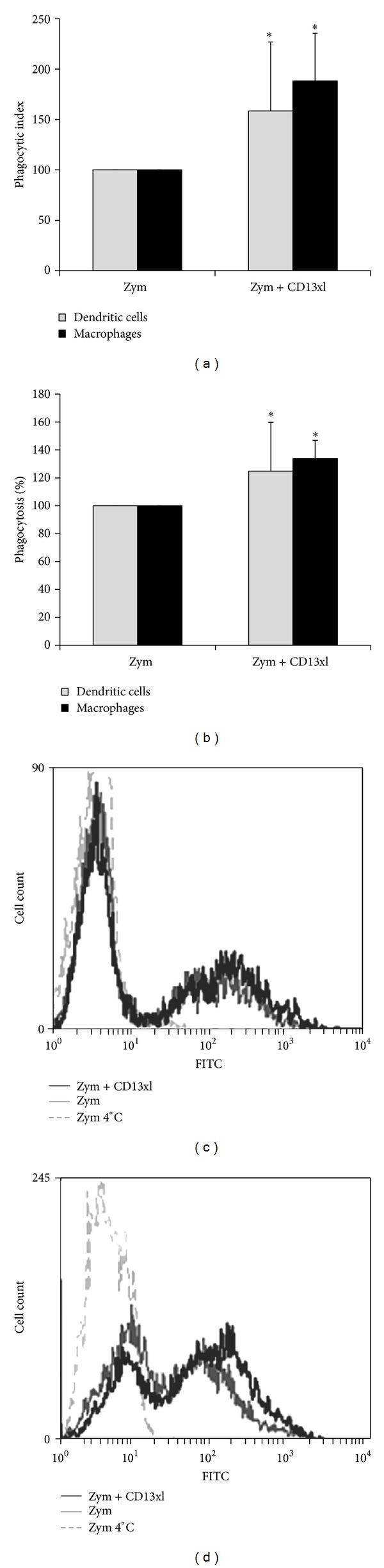
CD13 cross-linking enhances phagocytosis of zymosan by MD-DCs and MD-Ms. Cells were incubated with F(ab′)_2_ fragments of mAb 452 (anti-CD13) at 4°C. After washing, the cells were incubated with 20 *μ*g of zymosan-FITC in the presence (CD13xl) or absence of F(ab′)_2_ fragments of goat anti-mouse antibodies for 60 min (MD-DCs) or 30 min (MD-Ms) at 37°C. The phagocytic index (a) (geometric mean of fluorescence intensity multiplied by the percentage of positive cells) and the percentage of fluorescence positive cells (b) (% of phagocytosis) were determined using flow cytometry. Data were normalized considering the value obtained in the absence of CD13 cross-linking as 100%; *n* = 8 for MD-DCs and *n* = 6 for MD-Ms. *P ≤ 0.05. Histograms of a representative experiment with MD-DCs (c) and MD-Ms (d): dashed lines: cells incubated with zymosan at 4°C; gray lines: cells incubated with zymosan at 37°C; black lines: cells incubated with zymosan at 37°C in the presence of CD13 cross-linking. In this experiment, the percentages of CD14+ cells were 96% in MD-Ms and 16% in MD-DCs, and the percentages of CD1a+ cells were 18% in MD-Ms and 93% in MD-DCs.

Because of the high variability in the phagocytic indexes observed among cells from different individuals, the data of the phagocytosis of zymosan by each individual's cells were normalized, considering the value obtained in the absence of CD13 cross-linking as 100%. Analysis of the normalized data showed that in MD-DCs, the cross-linking of CD13 induced statistically significant increases of 25% in the percentage of phagocytic cells and of 58% in the phagocytic index of zymosan-FITC as compared to cells in which CD13 was not cross-linked (Figure 2). In MD-Ms, the potentiating effect of CD13 cross-linking on the phagocytosis of zymosan was more pronounced than in MD-DCs. In MD-Ms, CD13 cross-linking induced statistically significant increases of 88% in the normalized phagocytic index of zymosan-FITC and of 34% in the normalized percentage of phagocytic cells as compared to cells without CD13 cross-linking (Figure 2).

### 3.2. CD13 Cross-Linking Increases the Phagocytosis of *E. coli* by Human Monocyte-Derived Macrophages but Not by Dendritic Cells

**Figure 3:**
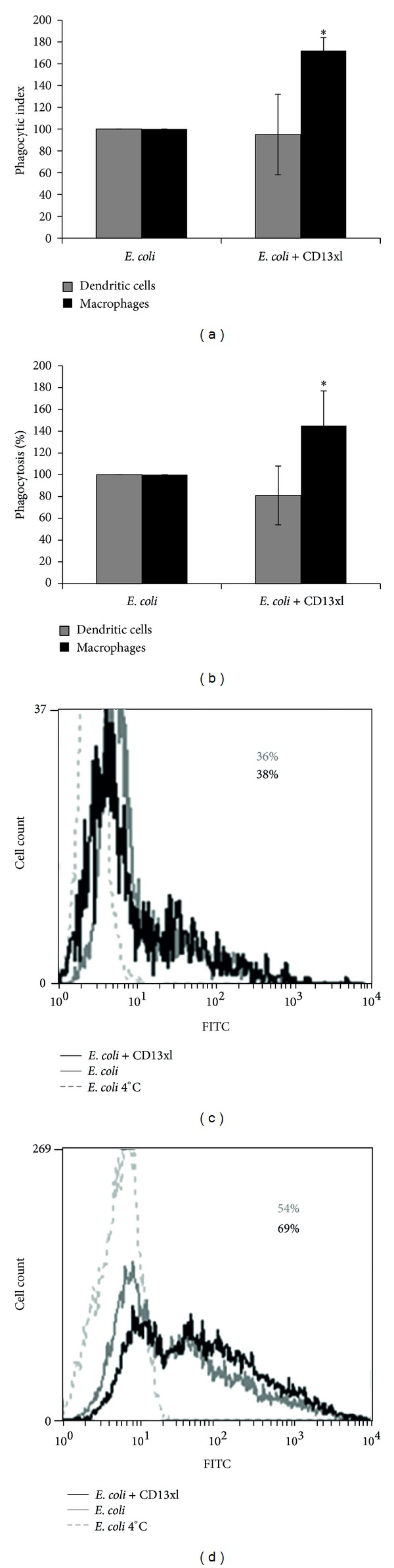
CD13 cross-linking enhances phagocytosis of *E. coli* by MD-Ms but not by MD-DCs. Cells were incubated with F(ab′)_2_ fragments of anti-CD13 mAb at 4°C. After washing, the cells were incubated with 12 *μ*g of *E. coli*-FITC, in the presence (CD13xl) or absence of F(ab′)_2_ fragments of goat anti-mouse antibodies for 60 min (MD-DCs) or 30 min (MD-Ms) at 37°C. The phagocytic index (geometric mean of fluorescence intensity multiplied by the percentage of positive cells) (a) and the percentage of fluorescence positive cells (% of phagocytosis) (b) were determined using flow cytometry. Data from each donor were normalized considering the value obtained in the absence of CD13 cross-linking as 100%. (a) and (b) show the averages of 6 experiments with cells from different donors, *P ≤ 0.05. (c) and (d) show representative histograms of an experiment with MD-DCs (c) and MD-Ms (d) from a single donor, after trypan blue; dotted lines: cells incubated with *E. coli* at 4°C; gray lines: cells incubated with *E. coli* at 37°C; black lines: cells incubated with *E. coli* at 37°C in the presence of CD13 cross-linking. The percentages of FITC+ cells were 36 and 38% for MD-DCs and 54 and 69% for MD-Ms without and with CD13 cross-linking, respectively.

We also evaluated the effect of CD13 cross-linking on the phagocytosis by MD-DCs and MD-Ms of nonopsonized FITC-labeled heat-killed *E. coli*. Both cell types internalized heat-killed bacteria, with phagocytosis percentages higher in MD-Ms (49.7 ± 22%) as compared to MD-DCs (31.7 ± 12%). After normalization of the data obtained from each individual donor's cells, we observed that, in MD-DCs, the cross-linking of CD13 did not induce significant effects on the phagocytic index, nor in the percentage of cells that ingested *E. coli* (Figure 3). In contrast, in MD-Ms, the cross-linking of CD13 induced statistically significant increases in the normalized phagocytic index (72% increase) and in the phagocytosis percentage (45% increase). Incubation of MD-Ms with F(ab′)_2_ fragments of anti-CD13 mAb 452, with no cross-linking or with F(ab′)_2_ fragments of goat anti-mouse antibodies only, had no effect on the phagocytosis of *E. coli*.

### 3.3. CD13 Is Internalized into the Surrounding Area of Ingested Zymosan Particles in Phagocytic Vesicles

**Figure 4:**
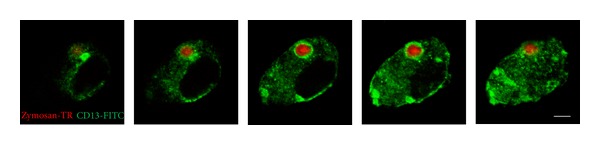
CD13 localizes at the phagosomal membrane after phagocytosis of zymosan particles in human monocyte-derived macrophages. Human MD-Ms were incubated with Texas Red-labeled zymosan particles for 30 min at 37°C. After washing away noningested particles, cells were fixed, permeabilized, and stained with anti-CD13 antibody and a secondary FITC-labeled goat anti-murine IgG. Samples were analyzed using confocal microscopy. From left to right: images of the same cell taken at 0.75 *μ*m intervals in the *z* axis. Bar: 5.0 *μ*m.

Since the data previously described showed that cross-linking of CD13 on cells' surface can potentiate the phagocytosis of zymosan and since we have previously reported that, in monocytes, CD13 is internalized into the phagosome during the phagocytosis of IgG-opsonized SRBCs, we determined the cellular localization of CD13 during phagocytosis of zymosan particles by MD-Ms. In order to avoid possible internalization induced by cross-linking of CD13 by specific antibodies, we first allowed MD-Ms to ingest TR-labeled zymosan particles. Then, after the phagocytosis assay, cells were fixed and permeabilized. The cellular distribution of CD13 was evaluated using anti-CD13 monoclonal antibody, followed by FITC-labeled anti-mouse IgG antibodies. Samples were analyzed using confocal microscopy. In resting conditions, CD13 was located both on cells' membranes and intracellularly. In cells incubated at 37°C with zymosan-Texas Red, we observed an important enrichment of CD13 in the immediate vicinity of the ingested particle (Figure 4). Interestingly, no evidence of colocalization (yellow-colored spots) was detected in the numerous samples analyzed. Control samples incubated at 4°C showed no internalized zymosan.

### 3.4. CD13 Mediates Particle Internalization in Human Monocyte-Derived Dendritic Cell

**Figure 5:**
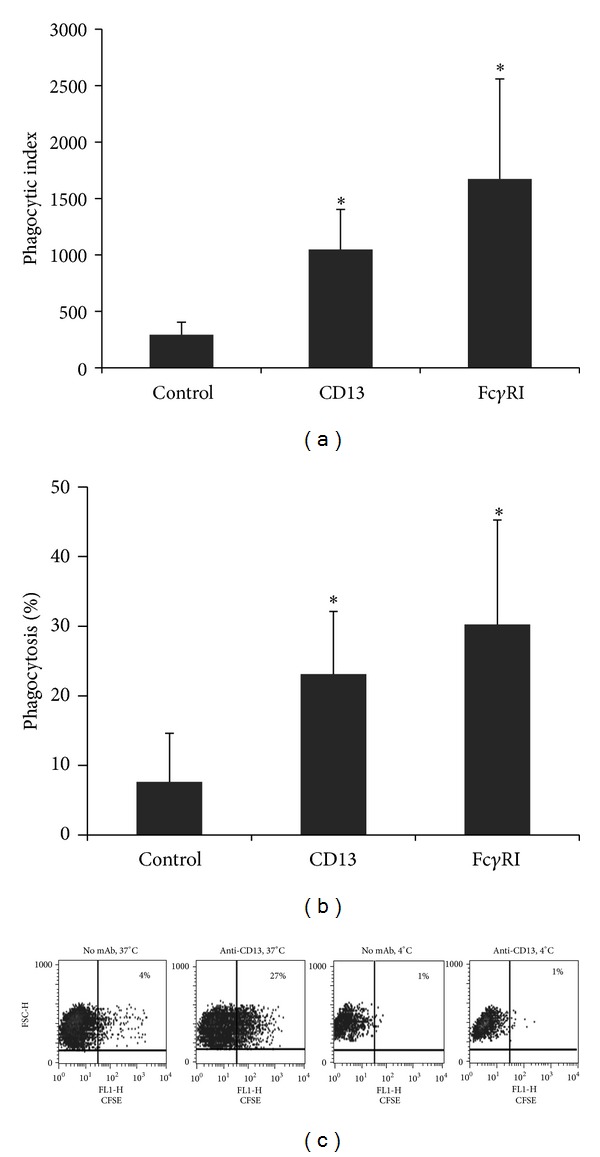
CD13 mediates internalization of particles in MD-DCs. MD-DCs were incubated with 2.5 *μ*g F(ab′)_2_ fragments of anti-CD13 mAb 452, with 2.5 *μ*g anti-Fc*γ*RI mAb 32.2, or with no antibody (control) for 30 min at 4°C. After washing, cells were mixed with CFSC-SRBCs previously sensitized with F(ab′)_2_ fragments of goat anti-mouse antibodies and incubated for 60 min at either 37°C or 4°C. Noningested SRBCs were lysed, and samples were analyzed using flow cytometry so as to determine the percentage and mean fluorescence intensity of positive cells. Phagocytic index was determined as indicated in Section 2. (a) and (b) show the average (±SD) phagocytic index and percentage of phagocytosis by MD-DCs obtained from monocytes of 8 different donors. *P ≤ 0.05. (c) shows representative dot plots of a single donor.

We had previously reported that human peripheral blood monocytes and U-937 monocytic cells can phagocytize SRBCs binding to monocytes through CD13 [12]. Because immature dendritic cells are known to be highly phagocytic, we evaluated whether CD13 can mediate phagocytosis in MD-DCs. In order to determine comparatively particle internalization mediated by CD13 and by a known phagocytic receptor (Fc*γ*RI), we employed a phagocytic assay in which the interaction of SRBCs with the phagocytic cell could be specifically directed to one or more cell receptors. Thus, we evaluated the internalization of CSFE-labeled SRBCs coated with F(ab′)_2_ fragments of goat anti-mouse antibodies, by MD-DCs previously incubated with F(ab′)_2_ fragments of anti-CD13 mAb 452 or anti-Fc*γ*RI mAb 32.2, as described in Section 2. The results show that, similarly to what was reported for monocytes, human MD-DCs are able to internalize SRBCs that bind to the cells' CD13. Although the percentage of phagocytic cells showed variations among cells from different donors, we consistently observed internalization of SRBCs through both CD13 and Fc*γ*RI (Figure 5). The average percentages (±SD) of cells with internalized SRBCs were 23 (±9), 30 (±15), and 7 (±7) for MD-DCs covered with CD13- and Fc*γ*RI-specific antibodies or lacking antibodies, respectively (*n* = 8). The average phagocytic index (percentage of phagocytic cells multiplied by the geometric mean of fluorescence intensity of the positive population) of MD-DCs from 8 different donors, incubated with anti-CD13 antibodies, anti-Fc*γ*RI antibodies, or with no antibodies, was 1047 ± 356 (CD13), 1673 ± 886 (Fc*γ*RI), and 292 ± 110, respectively. Both the phagocytic index and the percentage of positive cells in each individual donor were always higher for Fc*γ*RI than for CD13. Nevertheless, because of variations in the phagocytic capacity among cells from different individuals, no statistically significant difference was found between the phagocytosis mediated by the two different receptors (Figure 5). As expected, no significant internalization of SRBCs was observed in parallel samples of cells incubated with SRBCs at 4°C. These results show that human MD-DCs are able to ingest particles that bind to their membrane through CD13.

### 3.5. CD13 Cross-Linking Does Not Induce Cytokine Secretion, nor Does It Modulate TLR2-Mediated Cytokine Secretion in Human Monocyte-Derived Dendritic Cells or Macrophages

**Figure 6:**
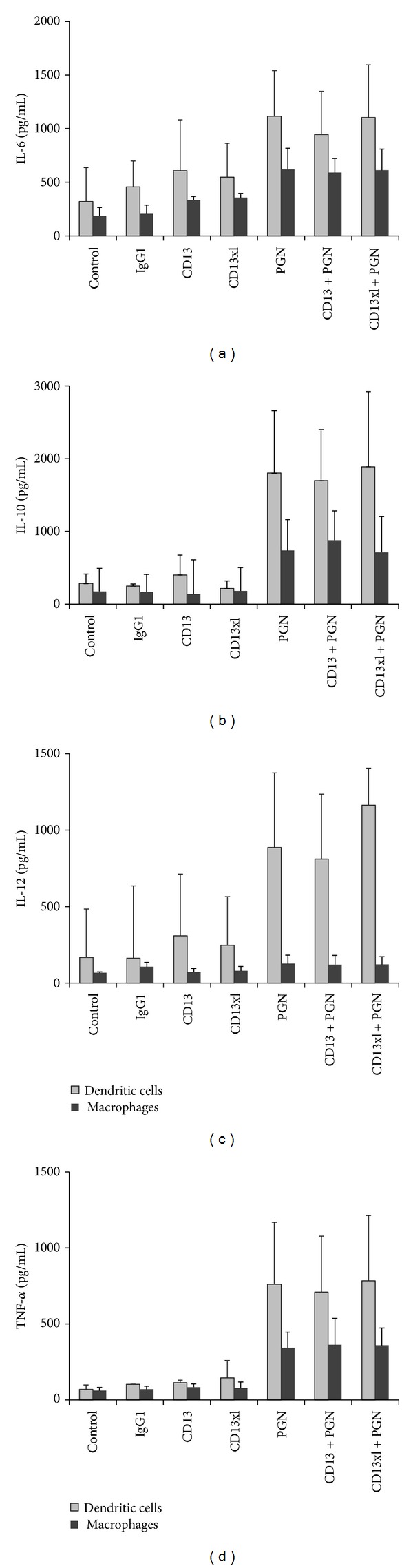
CD13 cross-linking does not modulate cytokine secretion induced by peptidoglycan from *S. aureus*. Human MD-DCs (gray bars) or MD-Ms (black bars) were stimulated with 10 *μ*g of anti-CD13 mAb 452, or with 10 *μ*g F(ab′)_2_ fragments of anti-CD13 mAb plus secondary antibody, in the presence or absence of 10 *μ*g PGN. After 18 h at 37°C, cells' supernatants were collected and assayed for the presence of IL-6, IL-10, IL-12, or TNF*α* using ELISA. Data shown are the average ± SD of three independent experiments with cells from three different donors.

Several pattern recognition receptors such as Dectin-1 are able to synergize with TLR2 through their activation of Syk kinase [17]. Since we had previously shown that CD13 is able to modulate Fc*γ*R-induced Syk activation [12], we decided to evaluate whether signaling through CD13 could affect TLR2-induced cytokine secretion in MD-DCs and MD-Ms. For that purpose, cells were incubated for 18 h with intact anti-CD13 mAb or their F(ab′)_2_ fragments plus a secondary antibody, and a known TLR2 ligand, peptidoglycan (PGN) from *Staphylococcus aureus *[18, 19]. IL-6, IL-10, IL-12, and TNF*α* secretion was determined in the supernatants. Treatment of MD-DCs with anti-CD13 antibodies induced no secretion of any of the cytokines analyzed, as compared to unstimulated cells or cells incubated with an isotype control antibody (Figure 6). When stimulated with PGN, the same MD-DCs produced significant amounts of those cytokines. Simultaneous cross-linking of CD13 did not induce a significant effect on secretion of cytokines induced by PGN. Similarly, in human MD-Ms, cross-linking of CD13 induced no secretion of any of the cytokines analyzed, nor did it affect cytokine secretion induced by PGN (Figure 6).

## 4. Discussion

Dendritic cells are specialized cells of the immune system that play an essential role in the onset of adaptive immune responses. On their membrane, dendritic cells express various innate (germline-encoded) receptors, through which they recognize and internalize pathogens and apoptotic cells. Thus, they become activated for secretion of cytokines, migration to secondary lymphoid organs, and antigen presentation for stimulation of naive T cells (reviewed in [20]). Receptors expressed by dendritic cells include TLRs, C-Type lectins, receptors for complement, Fc*γ* receptors, and scavenger receptors, among others. CD13 is highly expressed on the membrane of dendritic cells, monocytes, and macrophages, but the physiological relevance of its high expression in myeloid phagocytic cells is not fully understood yet. Nevertheless, some studies have reported the involvement of CD13 in specialized functions of myeloid cells. Thus, the participation of CD13 in extracellular trimming of antigenic peptides has been demonstrated. This trimming seems to be important for the T-cell stimulatory potency of the peptides, since inhibition of CD13 enzymatic activity by chemical inhibitors or anti-CD13 antibodies had a negative effect on the ability of peptide-MHC complexes to stimulate T cells [21–24]. It has also been shown that inhibition of CD13 enzymatic activity during *in vitro* differentiation of monocytes to dendritic cells blocks the development of immature DCs suggesting that CD13 plays a role in this differentiation process [25]. More recently, CD13 has been shown to regulate cross presentation of a soluble antigen (OVA) by CD8+ murine spleen DCs, negatively regulating antigen uptake by receptor-mediated endocytosis [3]. 

We have previously shown that, in human monocytes and macrophages, CD13 can modulate Fc*γ*R-mediated phagocytosis, since particles binding to the cell through Fc*γ*R and CD13 simultaneously were internalized more efficiently than particles bound through Fc*γ*R only [12]. In the present paper, we analyze whether CD13 could modulate phagocytosis mediated by receptors different from Fc*γ*Rs. We found out that phagocytosis of nonopsonized zymosan by MD-DCs and MD-Ms was significantly increased upon CD13 cross-linking with specific antibodies, and a similar enhancing effect of CD13 cross-linking was observed in the phagocytosis of heat-killed *E. coli* by MD-Ms. These results show that CD13 is able to modulate phagocytosis mediated by receptors other than Fc*γ*Rs. One of the main receptors for zymosan is Dectin-1 [26], although other receptors have also been suggested to participate in the phagocytosis of these particles, including scavenger receptors and a *β*-glucan receptor distinct from Dectin-1 [27]. In the phagocytosis of* E. coli*, various receptors have been suggested to participate, including C5aR [28] and CD14 [29]. Given the diversity of receptors involved in phagocytic processes that can be modulated by CD13, it seems that CD13 cross-linking positively modulates phagocytosis irrespective of the receptor involved, acting cooperatively with other receptors of the phagocyte. *In vivo*, this cooperative effect would depend on CD13 engagement either by a soluble molecule or by a ligand for CD13 on the surface of the particle. Although at present we cannot identify a CD13 ligand that could engage CD13 during *in vivo* phagocytosis of bacteria or other phagocytic preys, it is known that CD13 binds galectin-3 [30, 31], a beta-galactoside binding lectin, which is produced in substantial amounts by several cells—including macrophages—at inflammatory foci. Since galectin-3 is known to bind to several pathogens (for a review, see [32]), it can be proposed that secreted galectin-3 could act as an opsonin mediating the interaction of the pathogen with CD13. Of course, it is also possible that the large extracellular domain (925 residues) of CD13 contains as yet undiscovered binding sites for specific microbial components. 

In our previous experiments, the positive effect of CD13 on Fc*γ*R-mediated phagocytosis was observed during phagocytosis of particles bound to both CD13 and Fc*γ*R simultaneously. In contrast, the experiments reported in this paper show that a similar modulating effect is observed when CD13 is cross-linked physically separated from the binding of the particle to the phagocytic receptor. This suggests that the modulatory effect of CD13 on phagocytosis might be mediated by crosstalk between intracellular signaling pathways induced by the phagocytic receptors and signals induced by CD13 cross-linking. Although CD13 has a short cytoplasmic domain containing no signaling motif known, data from several groups indicate that CD13 cross-linking results in activation of signaling pathways. Santos et al. [33] demonstrated that, in monocytic cells, cross-linking of membrane CD13 by monoclonal antibodies is linked to the phosphorylation of MAP kinases such as ERK 1,2, JNK, and p38 and to an increase of intracellular [Ca^2+^]. Later, our group reported that co-cross-linking of CD13 with Fc*γ*RI by specific monoclonal antibodies increases the level and duration of Syk phosphorylation induced by Fc*γ*RI cross-linking [12]. Additionally, we have also reported that CD13 can be immunoprecipitated with the adaptors Grb-2 and Sos [34]. The mechanism by which CD13, with its short cytoplasmic tail, is able to induce intracellular signaling in monocytic cells, remains to be elucidated. Nonetheless, it is likely that this mechanism involves an association of CD13 with one or more auxiliary proteins, whose identity is unknown at present. The recent elucidation of the crystal structure of CD13 has shown that, in the dimer, each monomer can assume either an open or a closed conformation and that the conversion from the open/open dimer to the closed/closed dimer produces a significant conformational change that might be the basis of CD13 ability to produce intracellular signals, as this conversion produces a large change (~50 Å) in the distance between the membrane anchoring N termini of the two monomers in the dimer [35].

Results of both this study and our previous work [12] show that CD13 engagement positively modulates phagocytosis by different receptors in MD-Ms and MD-DCs. Nevertheless, since cells can efficiently internalize the same particles in the absence of CD13 engagement, CD13 is clearly not essential for phagocytosis. Thus, our results are not in conflict with the finding that mice with complete deletion of CD13 have no noticeable defects in Fc*γ*R- or scavenger receptor-mediated phagocytosis [14]. What we have shown here is that, when CD13 is cross-linked simultaneously, it can increase the phagocytosis of a variety of particles recognized by different receptors. The significance of this observation remains to be determined, but it might be important for an efficient phagocytosis when ligands' density on the surface of the particle is suboptimal.

Using confocal microscopy, we found out that, during the phagocytosis of zymosan, a significant fraction of CD13 is localized in the phagosome, surrounding the particle. This observation correlates with the data by Mina-Osorio and Ortega [12] where it was shown that CD13 redistributes to the phagosome in the phagocytosis of IgG-opsonized SRBCs by monocytic cell lines. It is remarkable that CD13 colocalizes in the phagosome containing the particles even though it is not known to interact with zymosan particles. We can speculate that, during phagocytosis mediated by certain receptors, CD13 is directed to the phagosome in order to perform some function, perhaps related to its enzymatic activity. The presence of CD13 in intracellular vesicles is in accordance with the findings of Gabrilovac et al. [13], who demonstrated that, in the murine macrophage cell line J774, CD13 colocalizes with mannose receptors (MR) and cointernalizes with the MR after the binding of its ligand (ovalbumin). Also, it has been reported that CD13 is internalized upon binding of some viruses [36] and that it plays a role in the uptake of cholesterol by the enterocyte brush border membrane [37].

Some reports have found out that CD13 might influence the production of cytokines by leukocytes. It was reported that two inhibitors of CD13 enzymatic activity (probestatin and actinonin) suppress IL-1*β* and IL-2 production in PBMCs and human T cells. These inhibitors also induce TGF-*β* production in PBMCs (reviewed in [38]). Lkhagvaa et al. reported that, in human monocytes stimulated with LPS, the CD13 inhibitor bestatin suppresses IL-6 and IL-8 production, while inducing IL-10 secretion [39]. In contrast, when CD4+ CD25+ T cells were stimulated with mitogens and treated with inhibitors of CD13, the production of TGF-*β* increased and the secretion of IL-2 decreased [40]. In our experiments, we observed no effect of CD13 cross-linking on the secretion of IL-6, IL-10, IL-12, or TNF*α* by cells stimulated by PGN, a TLR2 ligand. Also, CD13 cross-linking induced no cytokine secretion in MD-DCs or MD-Ms. These findings are in contrast with previous reports showing that treatment of U937 monocytic cells with an antibody specific for CD13 (clone WM15) produced an increase in the levels of mRNA for IL-8 [33]. It would be interesting to find out the reasons for this discrepancy. It is also important to continue characterizing the functions of CD13 in myeloid cells, as accumulating evidence suggests that this multifunctional protein might participate in specialized functions of that hematopoietic lineage.

## 5. Conclusions

We have shown that the cross-linking of CD13 positively modulates the phagocytosis of zymosan particles or heat-killed *E. coli* in human macrophages and dendritic cells. Also, during phagocytosis of zymosan, CD13 redistributes to the phagosome. These findings suggest that, besides its already known functions, CD13 also participates in phagocytic processes in myeloid cells.

## Abbreviations

APN: Aminopeptidase N
DC: Dendritic cell
MD-DC: Monocyte-derived-dendritic cell
MD-M: Monocyte-derived macrophages
Fc*γ*R: Receptor for the Fc portion of IgG
IL: Interleukin.
